# Evaluation of Aboveground Nitrogen Content of Winter Wheat Using Digital Imagery of Unmanned Aerial Vehicles

**DOI:** 10.3390/s19204416

**Published:** 2019-10-12

**Authors:** Baohua Yang, Mengxuan Wang, Zhengxia Sha, Bing Wang, Jianlin Chen, Xia Yao, Tao Cheng, Weixing Cao, Yan Zhu

**Affiliations:** 1 National Engineering and Technology Center for Information Agriculture/Jiangsu Key Laboratory for Information Agriculture/Collaborative Innovation Center for Modern Crop Production/Jiangsu Collaborative Innovation Center for the Technology and Application of Internet of Things, Nanjing Agricultural University, Nanjing 210095, China; 2 School of Information and Computer, Anhui Agricultural University, Hefei 230036, China; 3 School of Electrical and Information Engineering, Anhui University of Technology, Ma’anshan 243032, China; 4 Agricultural Information Institute of Science and Technology, Shanghai Academy of Agricultural Sciences, Shanghai 201403, China

**Keywords:** unmanned aerial vehicles, wheat, nitrogen concentration, camera, wavelet feature

## Abstract

Nitrogen (N) content is an important basis for the precise management of wheat fields. The application of unmanned aerial vehicles (UAVs) in agriculture provides an easier and faster way to monitor nitrogen content. Previous studies have shown that the features acquired from UAVs yield favorable results in monitoring wheat growth. However, since most of them are based on different vegetation indices, it is difficult to meet the requirements of accurate image interpretation. Moreover, resampling also easily ignores the structural features of the image information itself. Therefore, a spectral-spatial feature is proposed combining vegetation indices (VIs) and wavelet features (WFs), especially the acquisition of wavelet features from the UAV image, which was transformed from the spatial domain to the frequency domain with a wavelet transformation. In this way, the complete spatial information of different scales can be obtained to realize good frequency localization, scale transformation, and directional change. The different models based on different features were compared, including partial least squares regression (PLSR), support vector regression (SVR), and particle swarm optimization-SVR (PSO-SVR). The results showed that the accuracy of the model based on the spectral-spatial feature by combining VIs and WFs was higher than that of VIs or WF indices alone. The performance of PSO-SVR was the best (R^2^ = 0.9025, root mean square error (RMSE) = 0.3287) among the three regression algorithms regardless of the use of all the original features or the combination features. Our results implied that our proposed method could improve the estimation accuracy of aboveground nitrogen content of winter wheat from UAVs with consumer digital cameras, which have greater application potential in predicting other growth parameters.

## 1. Introduction

Nitrogen (N) is one of the essential nutrients for wheat growth. Accurate access to nitrogen information is a prerequisite for precise crop management and quality assurance [1,2]. Rapid and accurate access to nitrogen information in a non-destructive manner has become the primary means of wheat nutrition monitoring and management [3]. 

At present, nitrogen monitoring based on the remote sensing principle is receiving extensive attention [4,5]. Most of the data obtained are from remote sensing platforms, such as satellite remote sensing, airborne remote sensing, and spectrometers [6,7,8,9]. However, due to spatial resolution, spectral resolution, and temporal resolution, the remote data limit the value of agricultural applications and cannot meet the real-time requirements for crop growth monitoring [10,11,12]. In particular, light detection and ranging (LIDAR), hyperspectral, and multispectral sensors on unmanned aerial vehicles (UAVs) are not easily applied in practice due to their high price and complicated data processing requirements [13,14,15,16,17]. It could be seen that the fast, non-destructive, and high spatial resolution characteristics of UAVs have led agriculture to move toward quantitative refinement [18,19,20,21]. However, it remains unclear whether the estimation model for the N content of wheat can be improved based on the spatial-spectral relationship obtained from the same sensor without any additional cost for adding other hardware devices.

With the popularity of digital cameras and the development of digital image information acquisition and processing, crop monitoring estimates based on digital image technology have also been extensively studied. Such studies have shown that there are significant or extremely significant correlations between crop canopy parameters’ digital image from UAVs and biophysical parameters of crops, such as leaf area index (LAI) [22,23,24,25], biomass [26,27,28,29,30], nitrogen nutrition index [31], grain yield [32], and nitrogen content [33,34,35,36,37]. Although these studies have achieved good results, most of them are based on different spectral vegetation indices. However, the pure spectral features of pixels are insufficient to meet the requirements of accurate image interpretation [38]. In particular, spatial features are more important for digital image analysis from UAVs. How to quantitatively evaluate image spatial resolution from the image itself has always been a problem in the field of image processing. Studies have shown that ground-resolution images of 1, 2, 5, 10, 15, 20, 25, and 30 cm are recommended to estimate wheat biological parameters [39], whereas Lu et al. recommended 13.28 cm from comparisons using a series of pixel-sized images from UAVs, including 1.66, 3.32, 6.64, 9.96, 13.28, 26.56, 53.12, and 106.24 cm, for estimating wheat biological parameters [40]. It can be seen that the resolution of the image can only be quantitatively reduced (raised) by resampling, which can easily lead to the structural features of the image information being ignored. How to describe the spatial distribution feature at multiple scales is a very important task in remote sensing research. A wavelet transform can change the resolution of the image while maintaining the complete structural information of the image due to its good frequency localization feature, its scale transformation feature, and its direction-change feature [41].

The acquisition of digital images is essentially a process of signal scanning and digitization [42]. Therefore, it is crucial to extract the features of the image signal. At present, Fourier transform is used to decompose the image signal into sine waves of various frequencies [43]. However, the Fourier transform does not provide the characteristics of the signal in the time domain. Gabor transform is the process of convolving an image [44], even though this process cannot obtain satisfactory results for non-stationary signals. However, as a multi-scale analysis tool, wavelet transform provides a new idea for the extraction and analysis of spatial information due to its effective time–frequency positioning, which can decompose an image signal into a set of wavelets [45] and overcome the drawback of Fourier analysis. This can only describe information in a single band [46,47,48,49]. The wheat canopy images of different growth stages acquired by UAVs have different spatial structures. Therefore, to obtain the complete spatial information of different scales, it is necessary to use the wavelet transform to decompose the original signal of an unmanned aerial vehicle (UAV) image from the spatial domain to the frequency domain. To the best of our knowledge, there are few reports estimating nitrogen content in winter wheat using multi-scale spatial information.

Therefore, the study focuses on the feasibility of using a consumer-grade UAV to estimate the aboveground nitrogen content of wheat with a digital camera. For the use of the spatial-spectral features, the regression methods of partial least squares regression (PLSR) [50], support vector regression (SVR) [51], and particle swarm optimization-SVR (PSO-SVR) [52] were used to construct a model to meet the needs of precision agriculture. Therefore, the purpose of this study is to: (1) study the model performance of wavelet transform to estimate the aboveground nitrogen content of wheat, (2) combine the vegetation indices (VIs) and wavelet features (WFs) based on red, green and blue bands in order to improve the estimation model for aboveground nitrogen content of wheat, and (3) evaluate the performance of three regression techniques in the aboveground nitrogen content estimation model.

## 2. Data and Methods

### 2.1. Experimental Design

**Figure 1:**
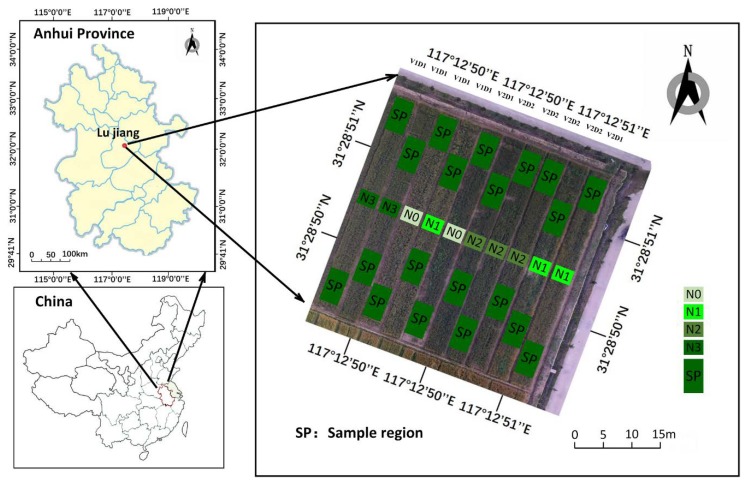
Location of the study area and the layout of field plots with wheat varieties, nitrogen levels, and sowing densities. Note: *SP* sampling region. D1 = 425 plants·m^−2^, D2 = 515 plants·m^−2^, N0 = 0 kg·N ·ha^−1^, N1 = 104 kg·N·ha^−1^, N2 = 150 kg·N·ha^−1^, N3 = 220 kg·N·ha^−1^, V1 = ‘Wanmai 55’, and V2 = ‘Ningmai 15’.

The experiments were carried out in 2019 at the National Agricultural Science and Technology Innovation and Integration Demonstration Base in Guohe Town, Lujiang County, Anhui Province. The area is located in the eastern part of Lujiang County (31.25° north latitude and 117.28° east longitude). Lujiang County is a subtropical monsoon climate zone with a humid climate, abundant rainfall, enough sunshine, and superior soil fertility. The average annual precipitation in this area is 995.3 mm. The average temperature is 16.8 °C. The annual maximum temperature is 36.4 °C and the lowest temperature is −4.0 °C. These factors are suitable for winter wheat growth. The experiments were carried out in 10 plots, with each plot being 168 square meters (42 × 4 m^2^). Four nitrogen levels were set including 0 (N0), 104 (N1), 150 (N2), and 220 (N3) kg/ha, of which 50% were used as a base fertilizer and 50% were added at the jointing stage. There were two planting densities (425 plants·m^−2^ and 515 plants·m^−2^) that were applied with three replications, as shown in Figure 1. The varieties were ‘Wanmai 55’ and ‘Ningmai 15’. The UAV remote sensing data acquisition experiment was carried out simultaneously with the field data acquisition and sampling. The growth period was obtained in three typical growth periods, which are flowering, filling, and maturity.

### 2.2. Data Collection

The UAV high definition (HD) digital images of the wheat breeding field were obtained in three key growth stages: flowering (9 May 2019), filling (14 May 2019), and maturity (24 May 2019) stages. The four-axis aerial vehicle UAV 3P (SZ DJI Technology Co., Shenzhen, China) was used to acquire images with a mass of 1280 g and unloaded flight lasted for about 23 min. The HD camera was installed on the drone remote sensing platform (Sony EXMOR 1/2.3 inches). The sensor (CMOS) pixel number was 12 million, the field of view was 94°, the focal length was 20 mm, and the aperture was f/2.8. Aerial photographs of the study site were taken from the UAV at a height of 40 meters above the ground. Every flight was carried out in a clear, cloudless, and windless weather and acquired about 43 images with a ground sample distance of 1.77 cm. The UAV was set to automatic flying mode. The side overlap and forward overlap of the image were set from 60% to 80%. The speed of UAV was 0.5 m/s. The international standards organization (ISO) of the digital camera was 100 and the best exposure was set depending on the weather conditions. The images were automatically captured, with one frame every 2 s, in a JPEG and DNG format. The same flight path and camera settings (excluding exposure) were used throughout the critical period of wheat growth.

The orthophoto maps were generated using Agisoft Photoscan 1.2.4 (Agisoft LLC, St. Petersburg, Russia) for the acquired images. This mainly included importing UAV images, aligning images, building a dense point cloud, building mesh, and generating orthophotos. The specific steps were as follows: first, the aerial image in the navigation band was selected, the feature point matching algorithm was used to automatically align the overlapping image, and then the “mild” depth was selected to construct the dense point cloud. Lastly, the image in the tagged image file format (TIFF) format was generated and further analyzed after the mesh with default parameters.

On the day after the UAV flight, 60 wheat plants were randomly cut as close to the soil surface as possible from each sample area in Figure 1. The wheat samples were taken back to the laboratory. All samples were placed in an oven at 105 °C for 30 minutes, and then dried at 80 °C for more than 20 h. They were then reweighed to obtain the dry mass of the winter wheat samples. The samples were pulverized. A Micro-Kjeldahl apparatus was used to measure the sample plant nitrogen concentration (SPNC, g·100g^−1^), and the aboveground nitrogen content (ANC, kg·ha^−1^) of winter wheat was determined by Equation (1) as follows.

(1) $$AGN=\frac{SPNC\times m\times n}{k}$$

where m is the dry mass of the winter wheat samples (kg·ha^−1^), k is the number of samples, and n is the number of winter wheat ears per unit area.

### 2.3. Feature Extraction

#### 2.3.1. Vegetation Indices

The VIs were the reflection and absorption characteristics of green vegetation in different bands. Vegetation information could be enhanced by combining different bands of sensors. The true color image acquired by the UAV included three bands of red, green, and blue, which include 700.0, 546.1, and 435.8 nm. In this study, based on the digital orthophoto map (DOM) of wheat, the average DN (digital number) values of the canopy red, green, and blue channels in each measured field were extracted, and the DN values of the three bands were defined as R, G, and B, respectively. Normalization was performed according to Equations (2)–(4) in order to reduce the effects of different illumination levels [53,54,55,56,57].

(2) $$r=\frac{R}{R+G+B}$$

(3) $$g=\frac{G}{R+G+B}$$

(4) $$b=\frac{B}{R+G+B}$$

In this case, G is the digital number of the green band of the feature, B is the digital number of the blue band of the feature, and R is the digital number of the red band of the feature. r, g, and b represent the digital numbers of the normalized red, green, and blue bands, respectively. A variety of color parameters could be derived from the combination of r, g, and b.

In this study, based on the DOM of three critical periods of wheat, the average DN values of the red, green, and blue channels of each measured area could be extracted. According to Table 1, 15 kinds of vegetation indices based on visible light bands were calculated. The vegetation indices and the corresponding aboveground nitrogen content of wheat formed a sample dataset. Additionally, 70% of the sample data was randomly selected as the calibration dataset, and correlation analysis was performed with the aboveground nitrogen content of wheat indicators of the simultaneously measured wheat to determine the sensitive vegetation indices.

#### 2.3.2. Wavelet Features

In order to extract multi-scale spatial information, wavelet features were obtained by a discrete wavelet transform [67]. The discrete transformation effect of the image was equivalent to the image undergoing a series of filtering transformations, including low-pass filtering (L) and high-pass filtering (H) [68]. In this study, two-dimensional discrete wavelet transform was used to decompose the image to form a pyramid structure. After decomposition, each layer produced one low-frequency subgraph and three high-frequency subgraphs in the horizontal, vertical, and diagonal directions, and the low-frequency subgraph was decomposed to obtain four wavelet subgraphs dissolved by the second layer. Thus, the low-frequency subgraphs generated by each layer could continue to be decomposed to generate the low-frequency subgraph and the high-frequency subgraph of the next layer. Therefore, the spatial information on different scales could be obtained, and the feature vectors were formed according to the scale order to realize the feature extraction of the region of interest (ROI).

The size of image I was M×N, the pixel of the image was (x,y), the wavelet coefficient of the image decomposition was $I_{ij}^{\Gamma }$, and the image was filtered by Equations (5)–(7).

(5) $$I_{xy}^{LH}=\frac{1}{N_{h}}\sum_{j=0}^{N_{h}-1}h(j)I_{\left(x/2-1)[(y+i-1)mod\;N\right]}^{L}$$

(6) $$I_{xy}^{HL}=\frac{1}{N_{l}}\sum_{i=0}^{N_{l}-1}l(i)I_{\left(x/2-1)[(y+i-1)mod\;N\right]}^{H}$$

(7) $$I_{xy}^{HH}=\frac{1}{N_{h}}\sum_{j=0}^{N_{h}-1}h(j)I_{\left(x/2-1)[(y+i-1)mod\;N\right]}^{H}$$

where l(i) $\left(i=0,1,2,\ldots ,N_{l}-1\right)$, h(j) $\left(j=0,1,2,\ldots ,N_{h}-1\right)$ are the impulse responses of the low-pass and high-pass filters and x=0,2,4…,M, y=0,1,2,…,N. $N_{l}$ and $N_{h}$ are the lengths of the low-pass and high-pass filters. LH, HL, and HH indicate the details of the horizontal, vertical, and diagonal directions, respectively [69].

The obtained high-frequency sub-images contained feature information in different directions. Therefore, the statistical values of wavelet transform coefficients were used to describe the image spatial features. In this paper, wavelet features were described according to Equations (8)–(11) with wavelet coefficients, including the mean, standard deviation, energy, and entropy [70].

(8) $$M=\frac{1}{MN}\sum_{i=1}^{M}\sum_{j=1}^{N}\left|I_{ij}^{\Gamma }\right|$$

(9) $$S=\frac{1}{MN}{\sum_{i=1}^{M}\sum_{j=1}^{N}\left(I_{ij}^{\Gamma }-C^{\Gamma }\right)}^{2}$$

(10) $$E=\frac{1}{MN}{\sum_{i=1}^{M}\sum_{j=1}^{N}\left|I_{ij}^{\Gamma }\right|}^{2}$$

(11) $$EN=-\frac{1}{MN}\sum_{i=1}^{M}\sum_{j-1}^{N}I_{ij}^{\Gamma }(\log2(I_{ij}^{\Gamma }))$$

where Γ=|LH,HL,HH|.

### 2.4. Methods

#### 2.4.1. Related Technologies 

It is important for multi-class remote sensing data to establish a relationship between remote sensing variables and crop parameters using regression techniques [71]. In this study, three regression techniques, which include PLSR, SVR, and PSO-SVR, were used to evaluate the performance of the aboveground nitrogen content model for winter wheat based on VIs, WFs, and spatial-spectral features from UAV images. PLSR is a common method of multiple regression analysis [72]. SVR can improve the generalization of learning, according to the principle of structural risk minimization, and reduce the empirical risk and confidence range [73]. Some parameters (penalty parameters, kernel parameters, and insensitive loss parameters) are more important for the SVR evaluation performance. Therefore, in this study, the particle swarm optimization (PSO) algorithm was used to optimize the selection of SVR parameters, so that the model based on PSO-SVR could obtain appropriate parameters to improve the accuracy of the model [74]. To improve the training efficiency of the model, principal component analysis (PCA) [75] and correlation coefficient analysis [76] were used to reduce the dimension of the variables, which were widely used in previous research.

#### 2.4.2. Model Validation

Correlation analysis is a statistical analysis method that studies the correlation between two or more variables [77]. The correlation coefficient (r) is often used to measure the closeness of the relationship between two data sets. In general, |r| is a number less than 1, and the larger |r|, the closer the relationship between the two sets of data. In this study, correlation analysis techniques were used to screen for characteristic variables that were closely related to the aboveground nitrogen content of wheat. In addition, to make the established model universal, the data were divided into a calibration set and a validation set according to the ratio of 7:3 in the experiment. The coefficient of determination (R^2^) and the root mean square error (RMSE) were used as the evaluation indicators of the model. All models constructed using regression techniques were validated and evaluated using Windows 10-based MATLAB R2017b (The MathWorks Inc., Natick, MA, USA).

The RMSE was used to evaluate the performance of the prediction. The correlation coefficient was used in all the processes. The RMSE was defined as follows.

(12) $${\mathrm{RMSE}}^{2}={\sum_{i=1}^{n}\left({\hat{y}}_{i}-\bar{y}\right)}^{2}/n$$

The correlation coefficient (R) was defined as follows.

(13) $$R^{2}=1-\sum_{i=1}^{n}{\left(y_{i}-\hat{y}\right)}^{2}/{\sum_{i=1}^{n}\left(y_{i}-\bar{y}\right)}^{2}$$

where n is the number of observations in the dataset, $\hat{y}$ and $y_{i}$ are the predicted and measured value of the *i*th observation, and $\bar{y}$ is the mean value of the calibration or validation set. 

## 3. Results and Analysis

### 3.1. Correlation Analysis between Vegetation Indices and Aboveground Nitrogen Content of Wheat

**Figure 2:**
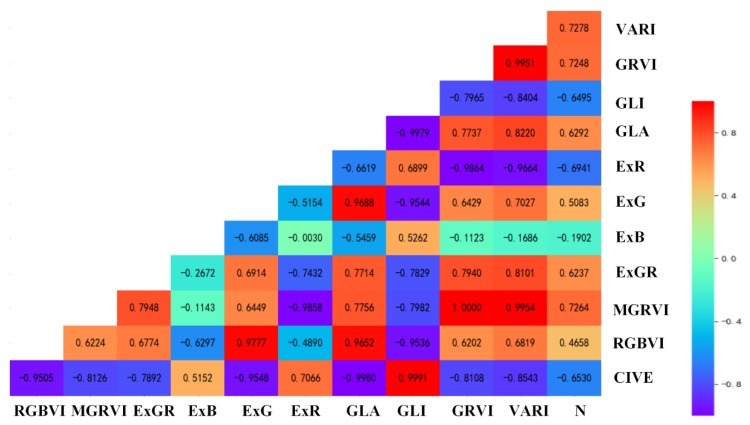
Matrix of the correlation coefficient between the N content and individual vegetation indices (VIs).

To explore the correlation between VIs and the aboveground N content of wheat, we combined the data of three periods, from which 70% of the data were randomly selected for correlation analysis. The results are shown in Figure 2. Most of the vegetation indices were strongly correlated with the aboveground nitrogen content of wheat. The absolute values of the correlation coefficients of VARI, MGRVI, GRVI, ExR, CIVE, GLI, GLA, ExGR, and the aboveground nitrogen content of wheat were between 0.6237 and 0.7278. These results are due to the top 50% of the strong correlation ranking. As a result, VARI, MGRVI, GRVI, ExR, and CIVE were selected as the optimal vegetation indices to build a subsequent model.

**Figure 3:**
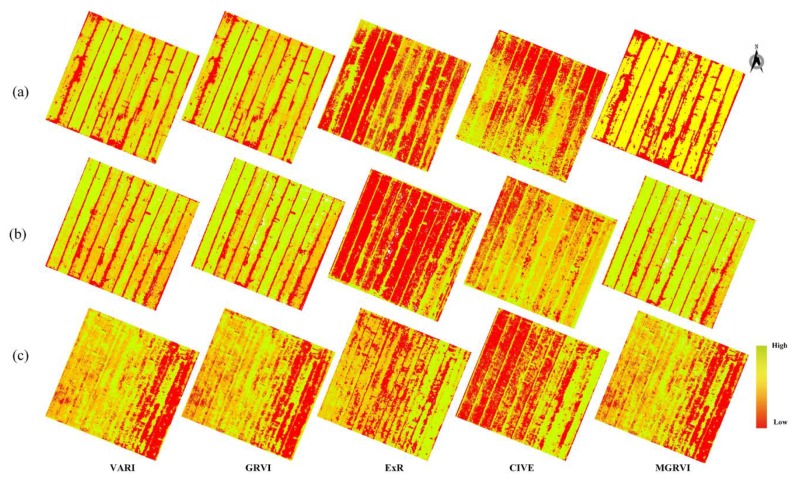
Spatial distribution of key vegetation indices at different periods. (**a**) Flowering, (**b**) Filling, and (**c**) Maturity stages.

Figure 3 shows the spatial distribution of VARI, GRVI, ExR, CIVE, and MGRVI for winter wheat in the study area at flowering, filling, and maturity stages. The different VIs in the same period were very different, and the same VIs varied with the growth period. ExR was relatively low in all three periods. During the filling period, VARI, GRVI, and MGRVI were relatively high. CIVE and MGRVI changed significantly in the three periods, and the other VIs were different in each period. Therefore, these five VIs could reflect the change of the aboveground nitrogen content of wheat, which has a certain representativeness.

### 3.2. Extraction and Analysis of Wavelet Features

**Figure 4:**
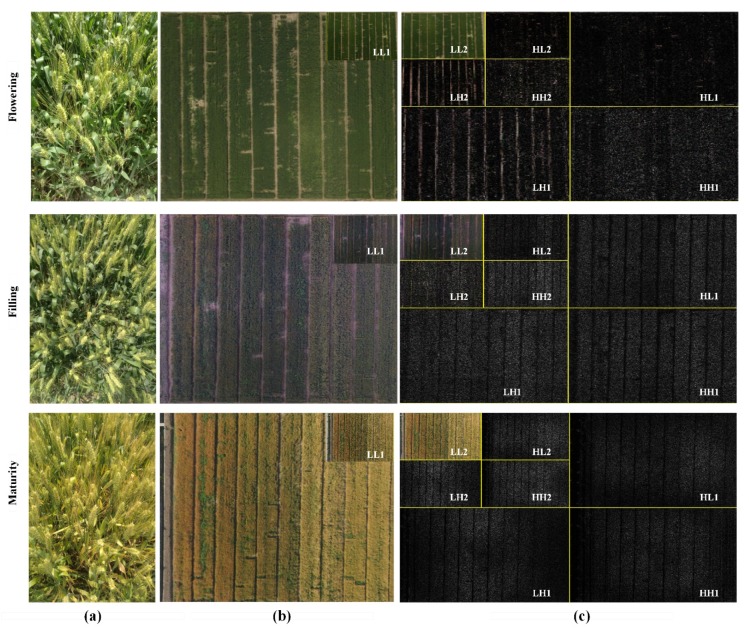
Two-layer wavelet decomposition based on images obtained by an unmanned aerial vehicle (UAV). (**a**) Canopy photos, (**b**) the original image and the low-frequency LL1 subgraph, and (**c**) a tower structure diagram of wavelet decomposition.

Daubechies was chosen as the wavelet basis function, and the horizontal and vertical filtering was used to realize the wavelet decomposition of the UAV image. Figure 4 shows the multi-resolution wavelet decomposition of the three critical periods of the wheat crop. Figure 4a shows the canopy image, and Figure 4b shows the original image and the second layer low-frequency wavelet decomposition subgraph. Figure 4c shows a high-frequency decomposition diagram of the tower, including two scales of high-frequency decomposition and low-frequency subgraph, wherein the first layer decomposition LL1 includes the main low-frequency information in the original image. LH1 represents the high-frequency information in the horizontal direction and HL1 represents the high-frequency information in the vertical direction. HH1 represents the high-frequency information in the 45° direction. When the low-frequency component LL1 is further decomposed by wavelet transformation, four bands of LL2, LH2, HL2, and HH2 as the second layer were obtained. It is clear that the greater the number of layers of wavelet decomposition, the lower the resolution of the decomposed wavelet subgraph. Therefore, only two wavelet decompositions were performed in this study.

**Figure 5:**
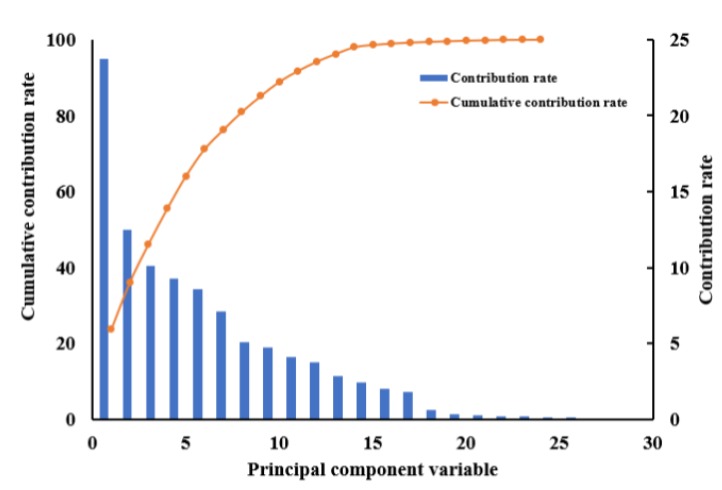
Contribution rate of the principal components.

For the wheat images of the three period samples, WFs were extracted from four subgraphs (LH1, HH1, HL1, LH2, HH2, HL2) of the high-frequency part of each layer, including the energy (E), entropy (En), mean (M), and standard deviation (S). As shown in Figure 4, a total of 24 WFs were obtained. First, statistical linear regression analysis was performed with the extracted WFs. Index values of most of the WFs were greater than 10, which showed that there was a severe collinearity between the independent variables. Moreover, the kaiser-meyer-olkin (KMO) statistic was 0.62 when the correlation between WFs was examined by the KMO test, which indicated that the correlation between the variables was strong and the partial correlation was weak. Furthermore, the result of the Bartlett test was less than 0.05, which demonstrated that the data were spherically distributed. From the above analysis, PCA was determined to be a suitable method for the further extraction of features. The results are shown in Figure 5. The contribution rate of the first eight principal components (PCs) reached 81.016%, which means only eight principal components could cover the information of 24 WFs. Among them, the contribution rate of PC1 (HL1E, LH1E, HL2E, LH2E, HH1E) was 23.693%, which mainly included the energy of the first layer and the second layer. The main contribution rate of PC2 was 12.460%, while the contribution rate of PC3 was 10.09% and the contribution rate of PC4 was 9.295%. The first four principal components occupied more than 50% of the total WFs.

### 3.3. Performance of Models Based on Different Methods

Three different variables, including VIs, WFs, and spatial-spectral features by combining VIs and WFs, were used as input variables for the estimation model, and aboveground nitrogen content was used as the dependent variable of the model. The PLSR, SVR, and PSO-SVR models were constructed to predict the aboveground nitrogen content of winter wheat. The results are shown in Table 2. The R^2^ of the calibration set and the validation set of the PLSR model range from 0.5618 to 0.7716, and the RMSE was below 0.8. The R^2^ of the SVR model ranged from 0.6483 to 0.8545. According to the parameters optimized by PSO, the PSO-SVR model was established, and the R^2^ of the calibration set and the validation set were both greater than 0.71. This showed that the regression techniques including PLSR, SVR, and PSO-SVR had strong predictive capabilities.

### 3.4. Performance Based on Different Feature Variables

**Figure 6:**
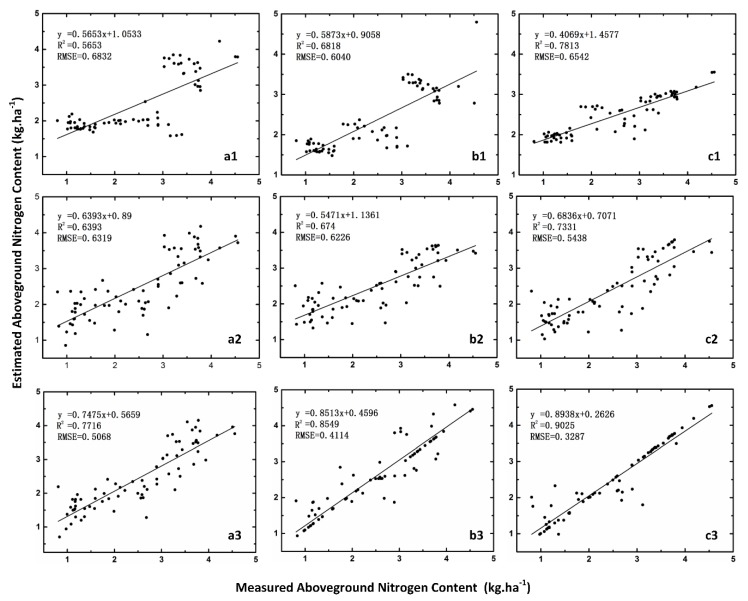
Estimated and measured winter wheat aboveground nitrogen content (kg·ha^−1^). Left: partial least squares regression (PLSR), Middle: support vector regression (SVR), right: particle swarm optimization-SVR (PSO-SVR) with vegetation indices (VIs) alone (**a1**,**b1**,**c1**), wavelet features (**a2**,**b2**,**c2**), and the combined data (**a3**,**b3**,**c3**). The data points displayed make up the validation set. The solid line indicates the fitting function of the scatter plot.

It was also found in Figure 6 that the models established based on different variables had significantly different effects. For instance, R^2^ ranged from 0.5653 to 0.7813 for VIs and 0.6393 to 0.7311 for WFs. The average RMSE of the calibration set is 0.64 and 0.59 kg·ha^−1^, respectively. 

The results of the estimated models using the three models of VIs and WFs were similar. The accuracy of the PLSR model using the integrated VIs and WFs was 26% higher than that of the VIs and 17% higher than that of the WFs, individually. The accuracy of the SVR model increased by 20% and 21%, and the accuracy of the PSO-SVR model increased by 13% and 19%, respectively. As shown in Figure 6, there was still a small amount of data in the model of WFs alone, which appeared under the fitted line. However, the data points in the model constructed by the integrated VIs and WFs were closer to the 1:1 line, which indicates that the model established using comprehensive indicators was better.

For VIs, the accuracy of the three models was not clear, which indicates that the model using only VIs was prone to saturation. However, the combination of VIs and WFs brought further improvements to all regression techniques, which are significantly more accurate than using VIs alone.

## 4. Discussion

### 4.1. Vegetation Indices and Wavelet Features 

Crop-growth monitoring based on the spectral information of crop canopies is significant [78]. Most traditional analysis methods are based on a single VI, which is used to construct a wheat N content prediction model, and good predictive models have been obtained. However, there is a certain spectral information saturation phenomenon that prevents the accuracy of the model from being improved [79]. In particular, the nitrogen of the vegetative organs of wheat shifts to the grain, which is accompanied by the decline of leaf photosynthetic performance and leaf senescence from the filling to the maturity stage. This causes the N content to decrease [80]. In addition, soil as a background causes changes in canopy spectral reflectance [81], which means that estimating the N-content based solely on VIs remains a challenge. Therefore, a spatial-spectral feature was proposed to improve the accuracy of the estimation model in this study. Compared with the Vis alone, the R^2^ value of the VIs and WFs as a spatial-spectral feature model increased by more than 17%, which indicates that the wavelet feature could weaken the saturation caused by spectral information. Therefore, the model constructed using the multi-feature parameters acquired by a UAV was more accurate, with the additional advantages being low-cost, having fast access to data, and requiring less computation.

### 4.2. Spatial Resolution and Wavelet Transform

The scale and resolution of the image acquired by the UAV were intrinsically linked, and the spatial resolution reflected the spatial detail level and the separation ability from the background environment. When the resolution of the wheat canopy image is too high, the internal spectral variability might increase, and the difference between crop characteristics might decline. When the resolution was too low, the mixed pixel phenomenon was serious, and the generated noise might affect the extraction of canopy features. The spatial resolutions were not uniform and were susceptible to light, canopy, and other factors. Moreover, estimating the resolution of the N content depended on the crop canopy size and line spacing [39]. Therefore, it is difficult to compensate for the lack of detailed multi-scale wavelet information, especially by only increasing the flying height of the drone or improving the resolution of the sensor.

In fact, the wavelet decomposition process is a filtering process with a characteristic energy concentration. The low-frequency part of the wavelet component is the approximation of the original image at different resolutions, and the high-frequency component includes details such as edges and contours. In this study, the wavelet decomposition of different layers indicates canopy images with different resolutions. Therefore, the image after a wavelet transform is equivalent to the process of resampling, which acquires not only the macroscopic structure but also the microstructure of the image. Thus, it could reduce or eliminate the influence of the soil background due to its anti-saturation property. In this study, only two layers of wavelet decomposition were performed. In future research, multi-layer wavelet decomposition based on other wavelet functions should be carried out to verify the feasibility of representing more than one spatial resolution.

### 4.3. Comparison of Feature Selection Methods

In the modeling process, most of the factors used for crop parameter inversion were directly input, which would affect the calculation speed and accuracy of the model. Good feature selection methods could improve the performance of the model, reduce the number of features, reduce dimensionality, and decrease over-fitting. Therefore, feature selection is very important, and it is necessary to use appropriate feature selection methods based on the relationship between different variables.

The Pearson correlation coefficient measures the linear correlation between variables, which can help us understand the relationship between features and variables. In this study, it was effective to use the correlation coefficient to analyze the importance of vegetation indices for N content. However, there were multiple correlations between some features, which worsened the generalization and stability of the model. Therefore, the principal component features extracted using PCA are more representative. In this study, eight principal components were extracted from 24 highly related wavelet features with PCA, and the results showed that the method was effective. Therefore, the feature selection method was not unique, but it was the most appropriate.

### 4.4. Comparison of PLS, SVR, and PSO-SVR for Estimating Aboveground Nitrogen Content 

Table 2 shows that the model accuracy of PLSR in the three models was the lowest, and there was also a nonlinear relationship between the feature parameters and the N content of wheat. The accuracy of the estimated model established by the machine learning method in this paper was always higher than that of the PLSR model. Moreover, the accuracy of the three models using only the WFs was generally not high. The accuracy of the PLSR model using the spatial-spectral feature was 15.8% higher than that using the WFs alone. The accuracy of the SVR model increased by 7.3% and 6%, and the accuracy of the PSO-SVR model increased by 12.6% and 17.2%, respectively. Whether a single feature or the spatial-spectral feature was used as an input variable to the model, PSO-SVR achieved the best calibration and validation precision in all three regression techniques.

Image feature extraction based on wavelet transform (Daubechies10 was employed as the wavelet basis in this study) can provide different technical approaches for using consumer-grade UAVs to predict the aboveground nitrogen content in different periods of winter wheat growth. A different wavelet basis function has its own features and scopes of application. Therefore, the determination of how to select the wavelet basis and decomposition scale still requires further study [82]. In addition, the model established in this study has the capability to resist interference. On the one hand, a wavelet transform can enhance the image. On the other hand, the wavelet can denoise the image due to its sparsity and multi-resolution characteristics [83]. Moreover, the construction and testing of the nitrogen-content monitoring model of winter wheat in this study is based on the field test data of an ecological area. Although the design of this experiment included different varieties, different planting densities, and different nitrogen fertilizer management strategies, the proposed method still achieved good prediction results. In the future, it is necessary to carry out extensive testing for improving different ecological points to further enhance the accuracy of model estimation, in order to promote the non-destructive diagnosis of crop nitrogen nutrition and precise fertilizer regulation. 

## 5. Conclusions

In this study, the VIs, wavelet features, and spatial-spectral features of images obtained from a UAV were used as input indicators, and models based on PLS, SVR, and PSO-SVR were constructed to estimate the aboveground N content of wheat. This provided a new method for the quantitative estimation of N content and realized the low-cost, rapid, and high-throughput monitoring of the wheat growth status and nutrition information for farmland irrigation. At the same time, it provided a guarantee for the fine management of factors such as farmland irrigation and fertilization.

The multi-scale canopy details were extracted by a wavelet transform to form spatial features of different scales. We proposed a wheat N-content estimation model based on spatial-spectral indicators, which was superior to the model estimation model based on VIs or WFs alone. This was mainly due to the combination of spectral-spatial features, including spectral information and image spatial information. Not only the spatial analysis of the features of different scales but also the decomposition of the signal from the image itself enabled us to overcome the influences of various factors during aerial photography. Therefore, the accuracy of the model significantly improved. Good results were obtained for estimating aboveground N with combined features with VIs and WFs using the PLSR model (R^2^ = 0.7171–0.7716, RMSE = 0.5068–0.5883), the SVR model (R^2^ = 0.7487–0.8545, RMSE = 0.4114–0.4841), and the PSO-SVR model (R^2^ = 0.797–0.9025, RMSE = 0.3287–0.4415). The results show that a drone equipped with a digital camera can improve the estimation of aboveground nitrogen content in winter wheat based on the obtained spatial spectral features. Moreover, various growth parameters of other crops, such as the leaf area index and chlorophyll content, could be effectively monitored in future studies.

## Figures and Tables

**Table 1:** Digital image variables related to the aboveground nitrogen content.

Indices.	Name	Formula	Reference
MGRVI	Modified Green Red Vegetation Index	$MGRVI=\left(g^{2}-r^{2}\right)/\left(g^{2}+r^{2}\right)$	[58]
RGBVI	Red Green Blue Vegetation Index	$RGBVI=\left(g^{2}-br^{2}\right)/\left(g^{2}+br^{2}\right)$	[58]
GRVI	Green Red Vegetation Index	GRVI=(g−r)/(g+r)	[59]
GLA	Green leaf algorithm	GLA=(2g−r−b)/(2g+r+b)	[60]
ExR	Excess Red Vegetation Index	ExR=1.4r−g	[61]
ExG	Excess Green Index	ExG=2g−r−b	[62]
ExB	Excess Blue Vegetation Index	ExR=1.4b-g	[63]
ExGR	Excess Green minus Excess Red	ExGR=ExG−ExR	[64]
CIVE	Color index of vegetation	CIVE=0.441r−0.881g+0.3856b+18.78745	[65]
VARI	Visible Atmospherically Resistant Index	VARI=(g−r)/(g+r−b)	[66]
GLI	Green Leaf Index	GLI=ExG/(−r−b)	[60]

**Table 2:** Estimation model for the aboveground nitrogen content of wheat based on different variables with three techniques.

Input Variables	Technique	Calibration		Validation	
		R^2^	RMSE (kg·ha^−1^)	R^2^	RMSE (kg·ha^−1^)
VIs	PLSR	0.5653	0.6832	0.5618	0.7917
	SVR	0.6818	0.604	0.6483	0.7176
	PSO-SVR	0.7813	0.6542	0.7132	0.7468
WFs	PLSR	0.6393	0.6319	0.6168	0.6596
	SVR	0.674	0.6226	0.6577	0.6009
	PSO-SVR	0.7311	0.5438	0.6962	0.6363
VIs and WFs	PLSR	0.7716	0.5068	0.7171	0.5883
	SVR	0.8545	0.4114	0.7487	0.4841
	PSO-SVR	0.9025	0.3287	0.797	0.4415

PLSR: partial least squares regression, SVR: support vector regression, PSO- SVR: particle swarm optimization- support vector regression, R^2^: coefficient of determination, RMSE: root mean square error, VIs: vegetation indices, WFs: wavelet features.

## Supplementary Materials

Supplementary File 1
