# Supplementary material for: Evaluation of Aboveground Nitrogen Content of Winter Wheat Using Digital Imagery of Unmanned Aerial Vehicles
Source: Sensors (Basel). 2019 Oct 12;19(20):4416. doi: 10.3390/s19204416 (PMC6832936; doi:10.3390/s19204416)
Supplement: Supplementary File 1 [file sensors-19-04416-s001.zip › Supplementary Files/English-editing-certificate-Baohua Yang.pdf]

We certify that the following article

Evaluation of Aboveground Nitrogen Content of Winter Wheat Using Digital Image of Unmanned Aerial Vehicle

Baohua Yang, Mengxuan Wang, Zhengxia Sha, Bing Wang, Jianlin Chen, Xia Yao, Tao Cheng, Weixing Cao, Yan Zhu *

has undergone English language editing by MDPI. The text has been checked for correct use of grammar and common technical terms, and edited to a level suitable for reporting research in a scholarly journal.
MDPI uses experienced, native English speaking editors. Full details of the editing service can be found at
► https://www.mdpi.com/authors/english.

MDPI Multidisciplinary Digital Publishing Institute

Basel, October 2019

Martyn Rittman, Ph.D.
English Editing Manager
englishediting@mdpi.com
https://www.mdpi.com/authors/english
